# Hidden costs of HIV treatment in Spain: inefficiency of the antiretroviral drug packaging

**DOI:** 10.7448/IAS.17.4.19609

**Published:** 2014-11-02

**Authors:** Josep M Llibre-Codina, Angels Andreu-Crespo, Gloria Cardona-Peitx, Ferran Sala-Piñol, Bonaventura Clotet-Sala, Xavier Bonafont-Pujol

**Affiliations:** 1 Hospital Universitari Germans Trias i Pujol, Fundació LLuita Contra la Sida, HIV, Barcelona, Spain; 2 Hospital Universitari GermansTrias i Pujol, Pharmacy, Barcelona, Spain

## Abstract

**Introduction:**

Antiretroviral drugs in Spain are delivered by law only in hospital pharmacies. Commercial packages meet variable quality standards when dispensed drugs are returned due to treatment changes or adherence problems Nearly 20–25% of the initial regimens will be changed at 48 weeks for different reasons. We evaluated the economic impact on public health system of the inability of using returned drugs due to inefficient packaging.

**Materials and Methods:**

We defined socially efficient packaging as the best adapted one to being delivered in unit dose to outpatients and classified: Class A - Drug packed in unit doses with complete info (name of drug, dosage in mg, lot, and expiring date) in each unit, maintaining complete information of the drug if returned when the external package is opened. Class B - packed in blisters with complete info in the blister, but not in unit doses, without special conservation conditions (should be re-packed in unit doses in the pharmacy before its dispensation to assure a class A excellence). Class C - packed in plastic containers with complete info written only on a label over the container, would allow repackaging only before its initial delivery, but not when returned. Class D - drug packed in plastic containers with manufacturer's warning that the product cannot be placed outside of the original package due to special conditions of conservation (fridge, humidity) that doesn't allow a unit dose repackaging or reusing an opened container. We analysed a 12-month period (July 2011–June 2012) in a hospital-based HIV outpatient pharmacy that serves 2413 treated individuals.

**Results:**

Patients generated 23,574 visits to pharmacy, and received 48,325 drug packages, with 2.529.137 pills delivered. The patients suffered 1051 treatment changes for any reason. A total amount of 122.945€ in treatment were returned to pharmacy in opened packages during the study period. 47.139.91€ would be totally lost, mainly due to being packaged in class C and D boxes, the equivalent of treating 78 patients with rilpivirine/TDF/FTC during 1 month. Class A and B packages in bad condition represented only 1.1% of the cost. However, 75.805€ came from returned packages in good condition that could potentially be reused. Most of the treatment changes were not foreseeable.

**Conclusions:**

A significant economic budget is lost through socially inefficient antiretroviral packages. Newer treatments are packaged in C and D categories, therefore maintaining these hidden costs in the near future. Any improvement in the excellence of packaging by the manufacturer, and favouring the choice of drugs supplied through efficient packages (when efficacy, toxicity and convenience are similar) should minimize the treatment expenditures paid by national health budgets.

